# Supplementary material for: A comprehensive catalogue of receptor-binding domains in extracellular contractile injection systems
Source: Nat Commun. 2026 Jan 22;17:1939. doi: 10.1038/s41467-026-68710-y (PMC12923769; doi:10.1038/s41467-026-68710-y)
Supplement: Supplementary file 1 — Supplementary Information [file 41467_2026_68710_MOESM1_ESM.pdf]

Supplementary Information

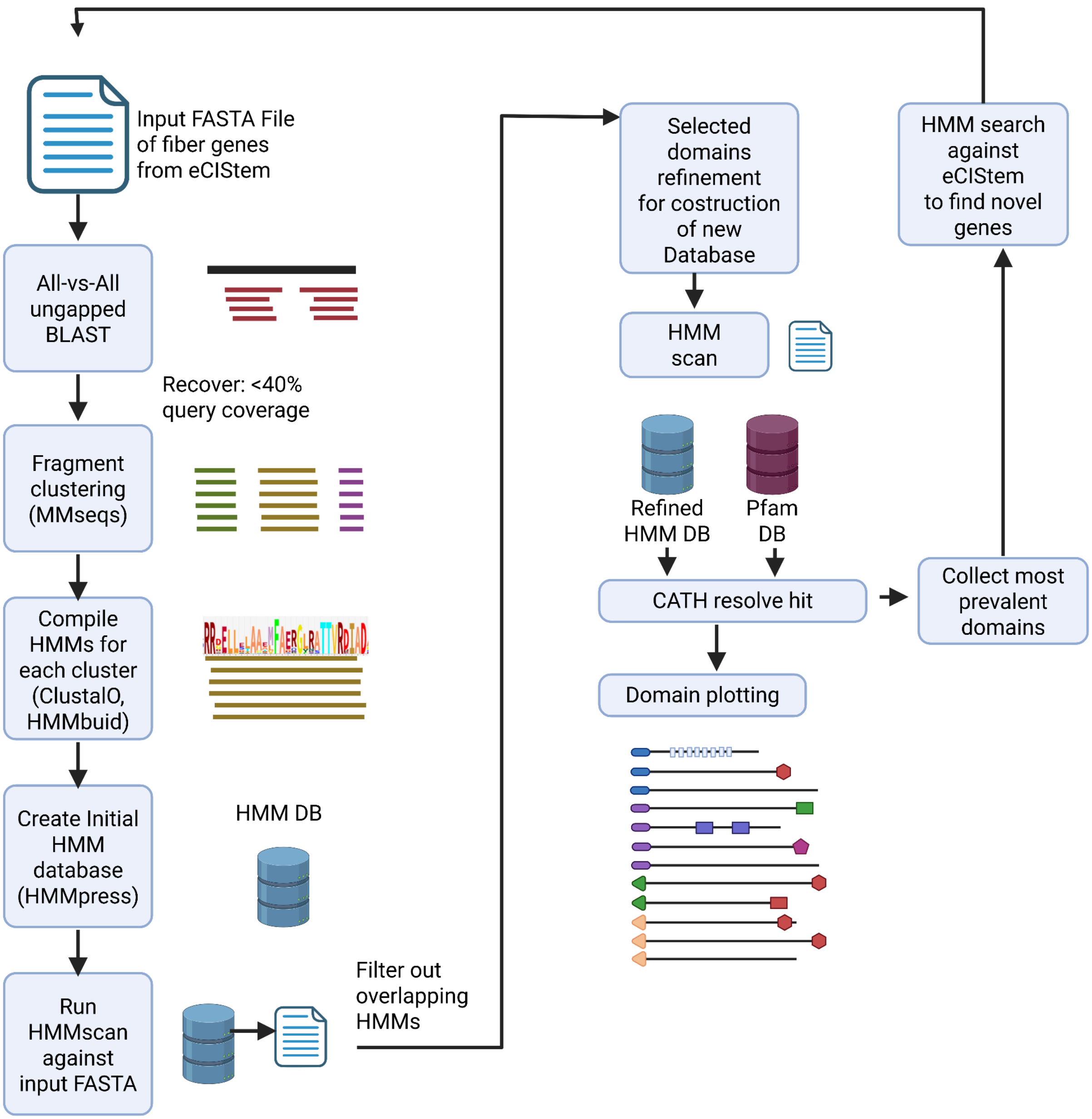

Supplementary Figure 1. Computational pipeline for the identification and characterization of eCIS tail fiber domains. Schematic representation of the domain discovery workflow used to identify and characterize eCIS tail fiber proteins. The pipeline consists of four main stages:
1. Initial sequence collection from the eCIStem database (1,425 operons) and identification of 629 putative fiber genes through similarity to known Afp13/Pvc13 homologs.

2. Domain detection phase using all-against-all BLAST followed by clustering (40-20% identity thresholds) with CD-HIT and MMseqs2.

3. Multiple sequence alignments were generated using Clustal Omega and converted to Hidden Markov Models (HMMs) with HMMER.

4. Dataset expansion using N-terminal conserved domains in jackhmmer iterative searches against our comprehensive microbial genome database, increasing coverage to 3,445 fiber genes across 2,585 operons. The figure was created in BioRender. Levy, A. (2025) https://BioRender.com/q2rg02f

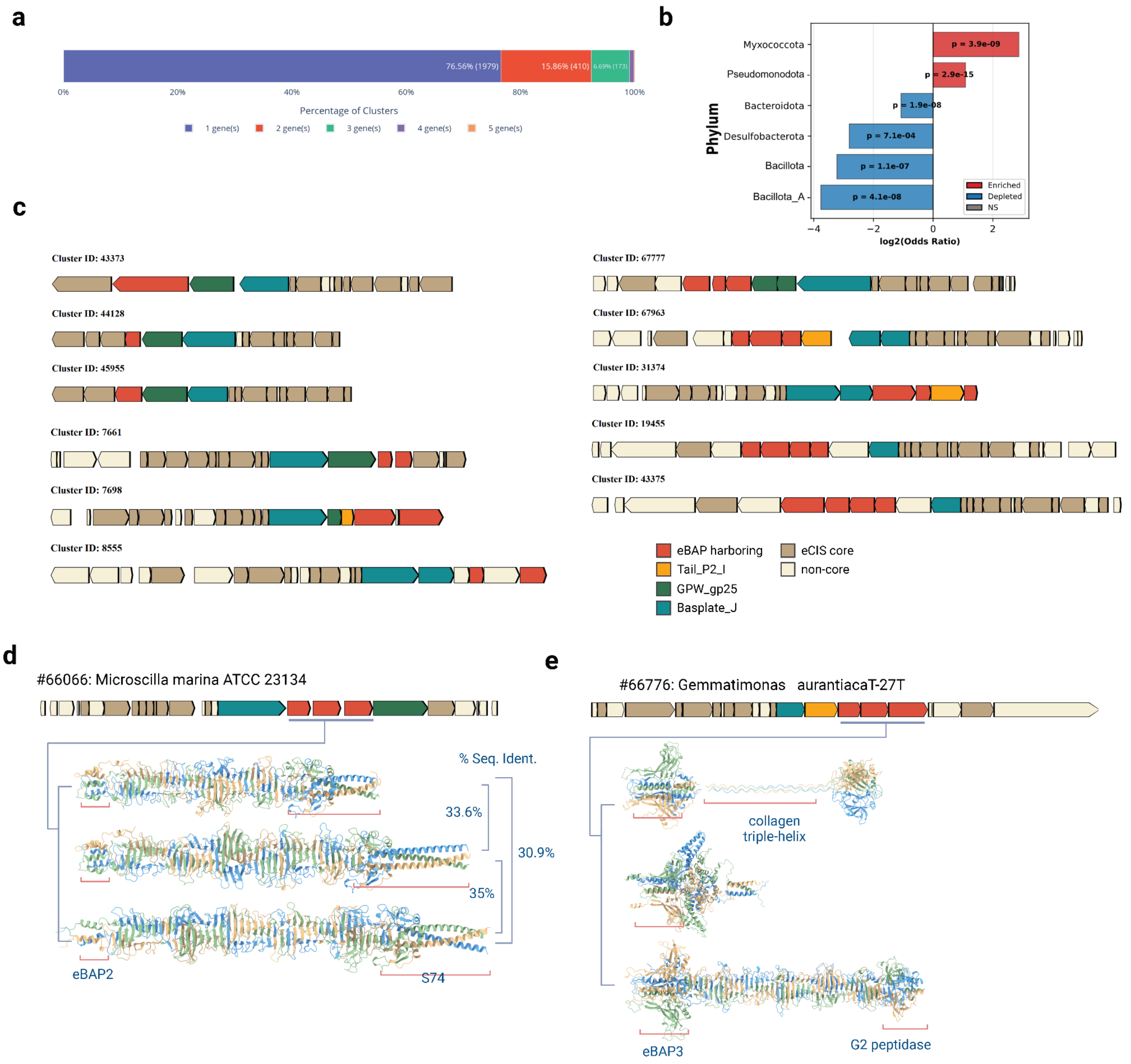

Supplementary Figure 2. Genomic organization and evolutionary patterns of multi-fiber eCIS operons

(a) Stacked bar chart showing the distribution of fiber gene counts per eCIS operon across the dataset (N=2,585 operons). Nearly one-quarter of operons (23.4%) encode multiple tail fibers, with a maximum of five fiber genes per locus.

(b) Enrichment analysis for phyla enriched with multi-fiber eCIS loci. Represented as diverged bar-plot with p-value written on the bars. Red bars show the enrichment pattern in *Myxococcota* and *Pseudomonodota.* The rest are either non-significant or completely depleted.

(c) Representative genomic neighborhoods of eCIS operons containing 1-5 fiber genes. Fiber genes (deep red) consistently localize downstream of conserved baseplate components (AFP11\12 identified by Baseplate_J and GPW Pfams respectively and the tail_P2_I harboring genes).

(d) Example operon containing three structurally similar fiber genes with high sequence divergence (maximal 35% pairwise sequence identity). AlphaFold2 predictions reveal conserved eBAP2 with similarly looking fibers with C-terminal S74 domains.

(e) Operon encoding three architecturally distinct fibers: a common eBAP3 domain found on three fibers with diverged domain architectures.

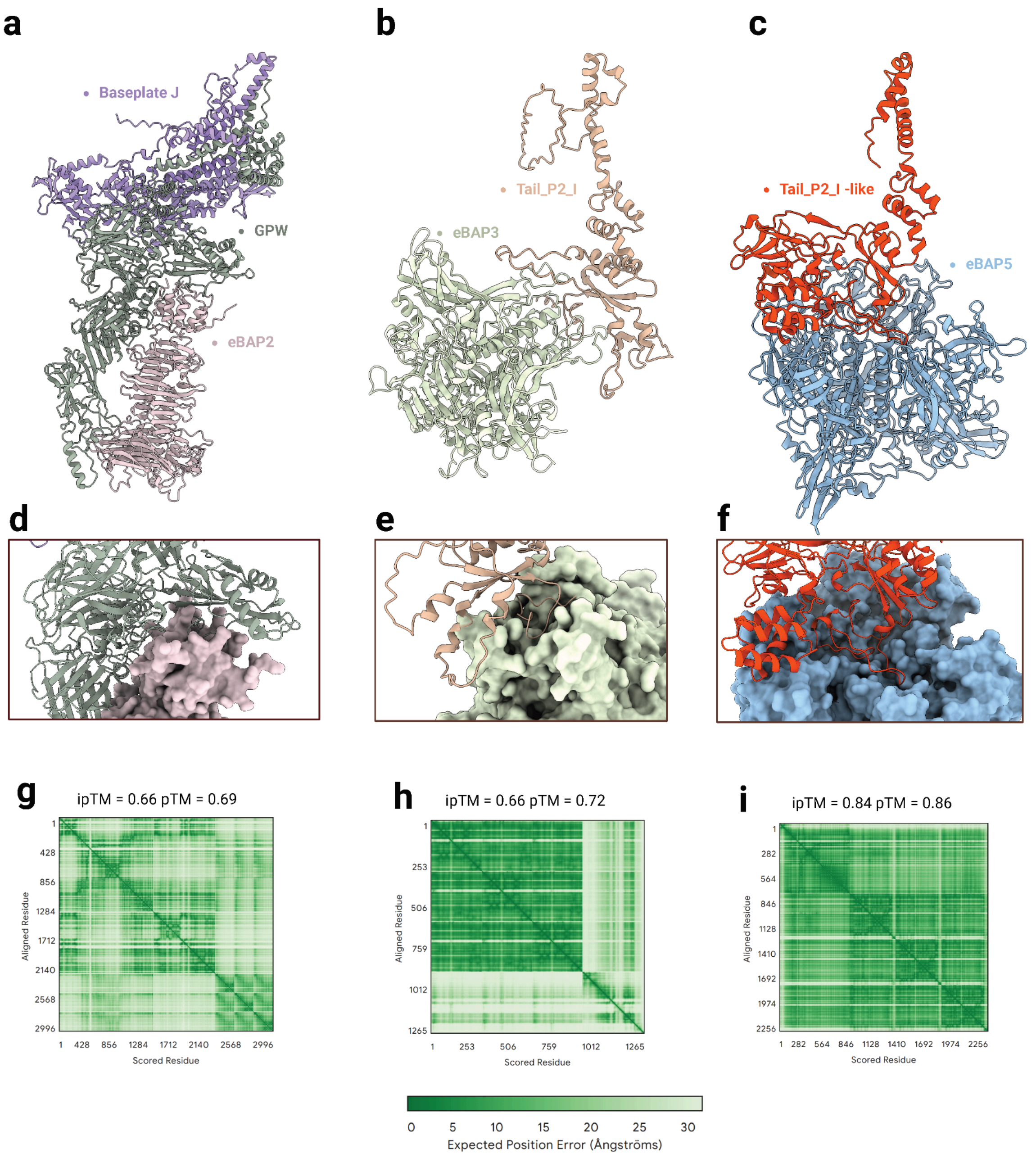

Supplementary Figure 3. Structural basis of eBAPs fiber attachment to eCIS baseplate components

(a-c) Predicted trimeric structure of unexplored eBAPs with baseplate components: **a.** the AFP11-homolog (baseplate_J protein) (olive green) in a structural complex with eBAP2 fiber N-terminal domains (pinkish).

(b). eBAP3 domain in complex with Tail_P2_I harboring gene **c**. eBAP5 domain in complex with upstream gene structurally similar to Tail_P2_I protein

(d-f) Zoomed view of the eBAPs baseplate interface. Key interactions include: eBAPs are predicted to interact with loops stemming from base plate proteins

(g-i) ipTM, pTM and Predicted Aligned Error (PAE) plots from AlphaFold3 multimer predictions.

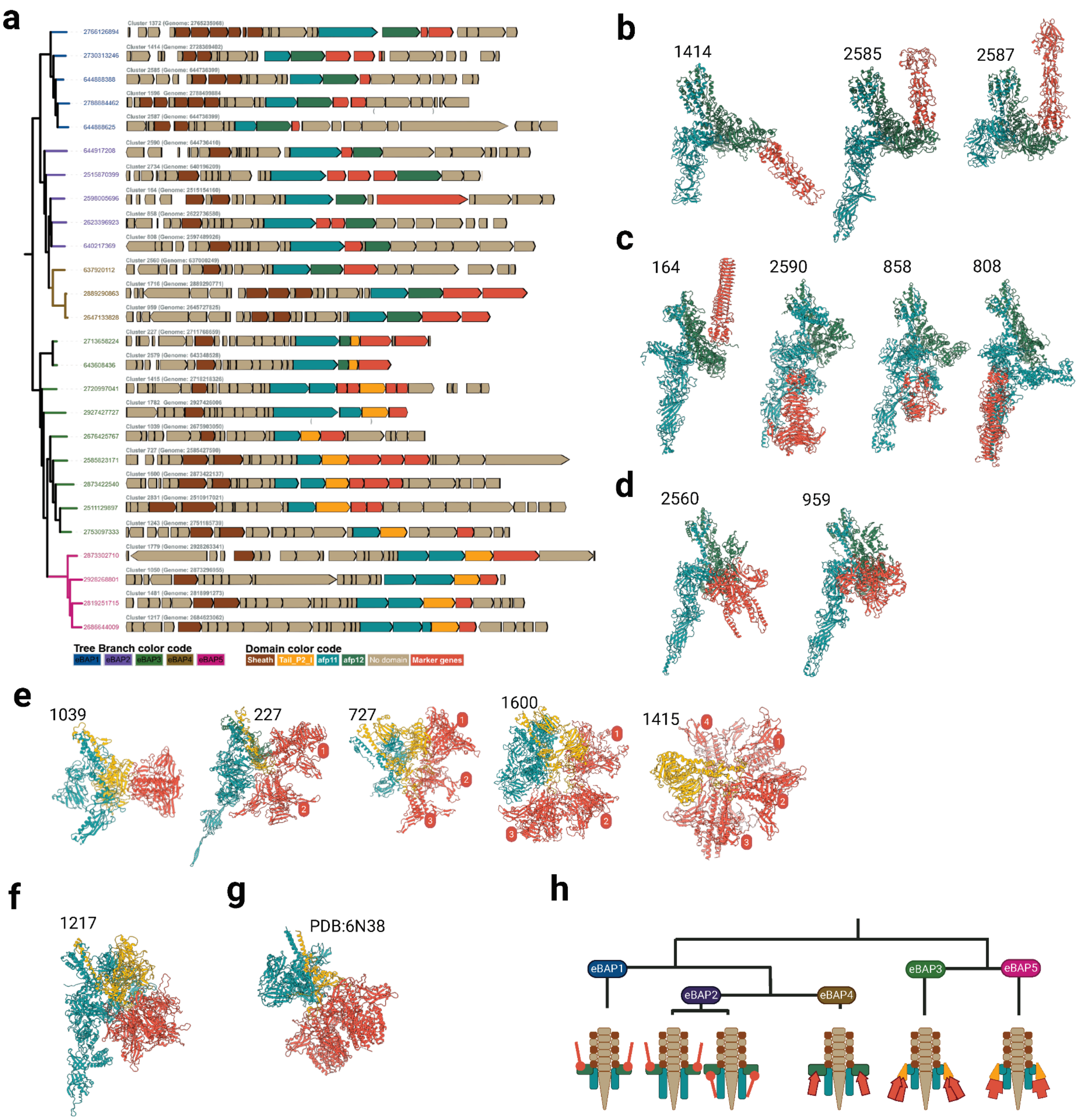

Supplementary Figure 4. Phylogenomics and Structural Analyses Reveal Two Main Baseplate Types Driving Distinct eCIS Evolutionary Trajectories.

(a) Phylogenetic tree of Afp11 sequences combined with genomic neighborhood analysis showing clustering patterns congruent with eBAP domain distributions. Tree branches are colored by eBAP type, with two distinct baseplate architectural groups highlighted: Group I (eBAP1, 2, 4) containing both Afp11/12 homologs but lacking Tail_P2_I genes, and Group II

(eBAP3, 5) relying exclusively on Tail_P2_I adaptors and largely lacking Afp12 homologs. Bootstrap values >70% are shown at major nodes.

(b-d) Structural modeling of eBAP assembly architectures using AlphaFold2-multimer predictions. **(b-c)** eBAP1-2 fibers showing backward tilt configuration similar to Afp/PVC systems. **(d)** eBAP4 anchoring at the baseplate bottom resembling tCIS architecture. **(e-f)** eBAP3,5 systems utilizing loop-mediated attachment through Tail_P2_I components, capable of accommodating up to four fibers per baseplate.

(g) T6SS baseplate architecture as displayed in PDB structure 6N38. Structural homology is inferred using the same color codes as used for eCIS baseplate proteins.

(h) Schematic model illustrating the dual baseplate architecture paradigm driving eCIS structural diversification. The two evolutionary trajectories are represented by distinct baseplate compositions and fiber attachment mechanisms, with Group I utilizing Afp11/12-mediated anchoring and Group II employing Tail_P2_I-based loop-mediated attachment systems. Created in BioRender. Levy, A. (2025) https://BioRender.com/q2rg02f .

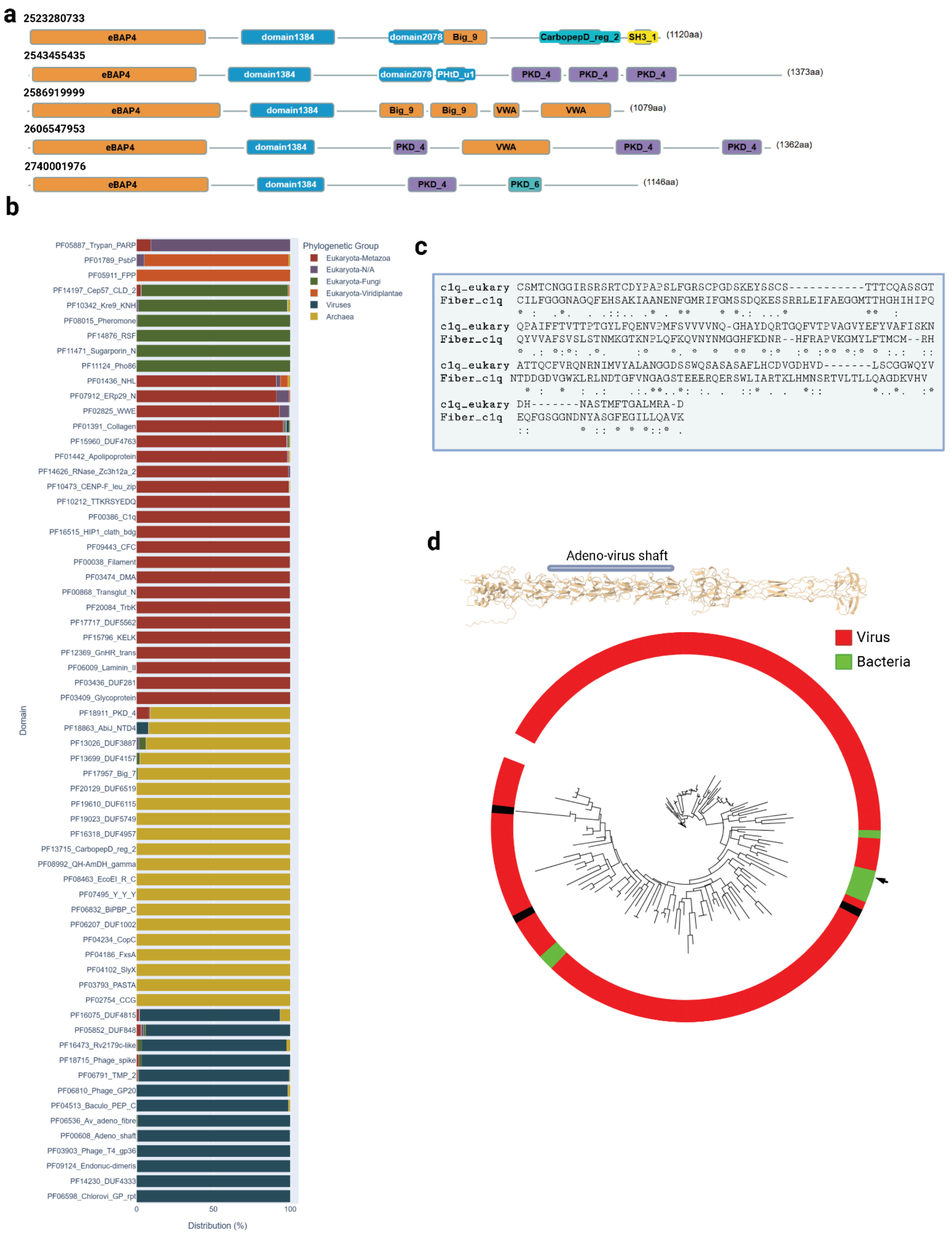

Supplementary Figure 5. Evidence suggesting acquisition of domains in eCIS tail fibers

(a) Domain architecture of eBAP4 genes displays a mix of Ig-like domains ordered in different combinations of c-terminal chains. Domains detected on each gene by Pfam hmmscan displayed as domain architectures ordered from left to right (on amino acid sequence from N' to C' termini respectively). Gene ID displayed above each line.
(b) Taxonomic distribution of 64 Pfam domains showing phylogenetic incongruence across kingdoms. Stacked bars represent the relative share of Pfam group members from Bacteria (), Eukaryota-Metazoa (Dark red), Eukaryota-N\A (no detectable subgroup, purple), Eukaryota-Fungi (Olive green), Eukaryota-Viridiplantae (orange), Viruses (Navy blue) and Archaea (Yellow).These domains are with >90% non-bacterial representation suggest horizontal gene transfer events.
(c) C1q domains MSA from figure 3g.
(d) Adenovirus shaft homolog analysis. Top: Structural alignment demonstrating conservation of the triple β-spiral fold and key proline residues that maintain characteristic bend angles across viral and bacterial domains. Bottom: Phylogenetic tree showing bacterial eCIS fiber shaft domains clustering with viral sequences rather than forming a separate bacterial clade.

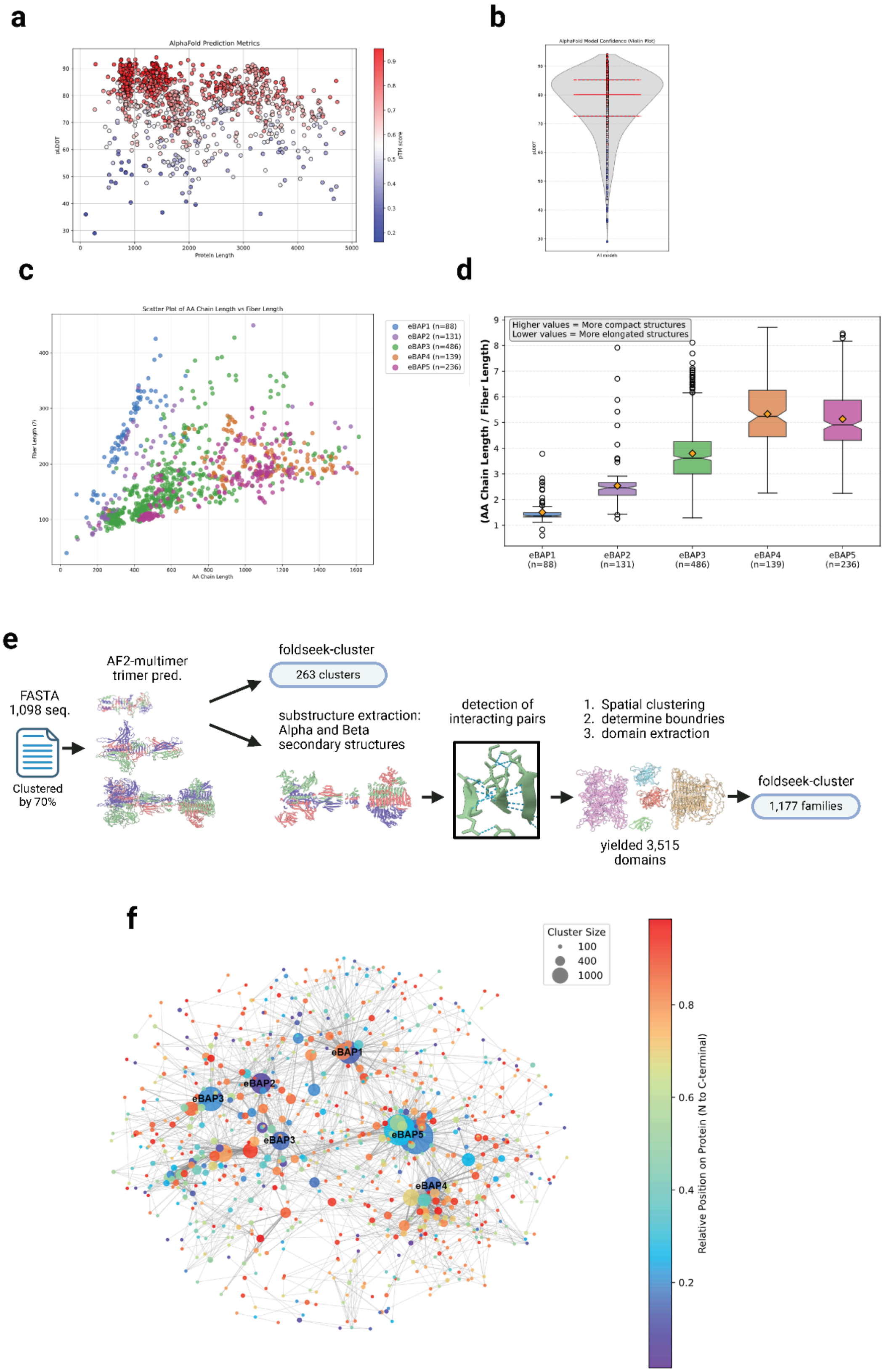
a
AlphaFold Prediction Metrics
pLDDT
Protein Length
pTM score
b
AlphaFold Model Confidence (Violin Plot)
c
Scatter Plot of AA Chain Length vs Fiber Length
eBAP1 (n=88)
eBAP2 (n=131)
eBAP3 (n=486)
eBAP4 (n=139)
eBAP5 (n=236)
AA Chain Length
d
Higher values = More compact structures
Lower values = More elongated structures
(AA Chain Length / Fiber Length)
eBAP1 (n=88)
eBAP2 (n=131)
eBAP3 (n=486)
eBAP4 (n=139)
eBAP5 (n=236)
e
FASTA
1,098 seq.
Clustered by 70%
AF2-multimer trimer pred.
foldseek-cluster
263 clusters
substructure extraction:
Alpha and Beta secondary structures
detection of interacting pairs
1. Spatial clustering
2. determine boundries
3. domain extraction
yielded 3,515 domains
foldseek-cluster
1,177 families
f
Cluster Size
100
400
1000
eBAP1
eBAP2
eBAP3
eBAP3
eBAP5
eBAP4
Relative Position on Protein (N to C-terminal)
0.2
0.4
0.6
0.8

Supplementary Figure 6. Structural prediction database parameters and domain analysis

(a) Scatter plot of pLDDT and pTM structure quality parameters laid out by protein chain length.

(b) Quantitative violin plot of parameter in (a) displaying the overall pLDDT distribution.

(c) Scatter plot of fiber bulkyness analysis and estimation. This was done by comparison of length of predicted structure (in angstrom) vs the length of the amino-acid chain.

(d) Quantitative aspect of (c) using box plot.

(e) Schematic representation of the structural pipeline. We used a fasta database clustered by 70% similarity and coverage. Predicted trimeric structures of >1000 proteins. Used in two pathways. 1. Clustering of whole fibers. 2. Domain dissection via 3D models: secondary structures were extracted from PDB files we analyzed interactions between secondary structures by the rule of one closest residue. We clustered secondary structures in order to define domain boundaries. This resulted in 1,177 domain foldseek clusters. Created in BioRender. Levy, A. (2025) https://BioRender.com/q2rg02f . **(f)** We generated a network of domain foldseek clusters with node size representing domain abundance. Coloring represents domain relative position on protein linear sequence. Edges are drawn if the clusters contain the same protein ids implying for shared architectures.

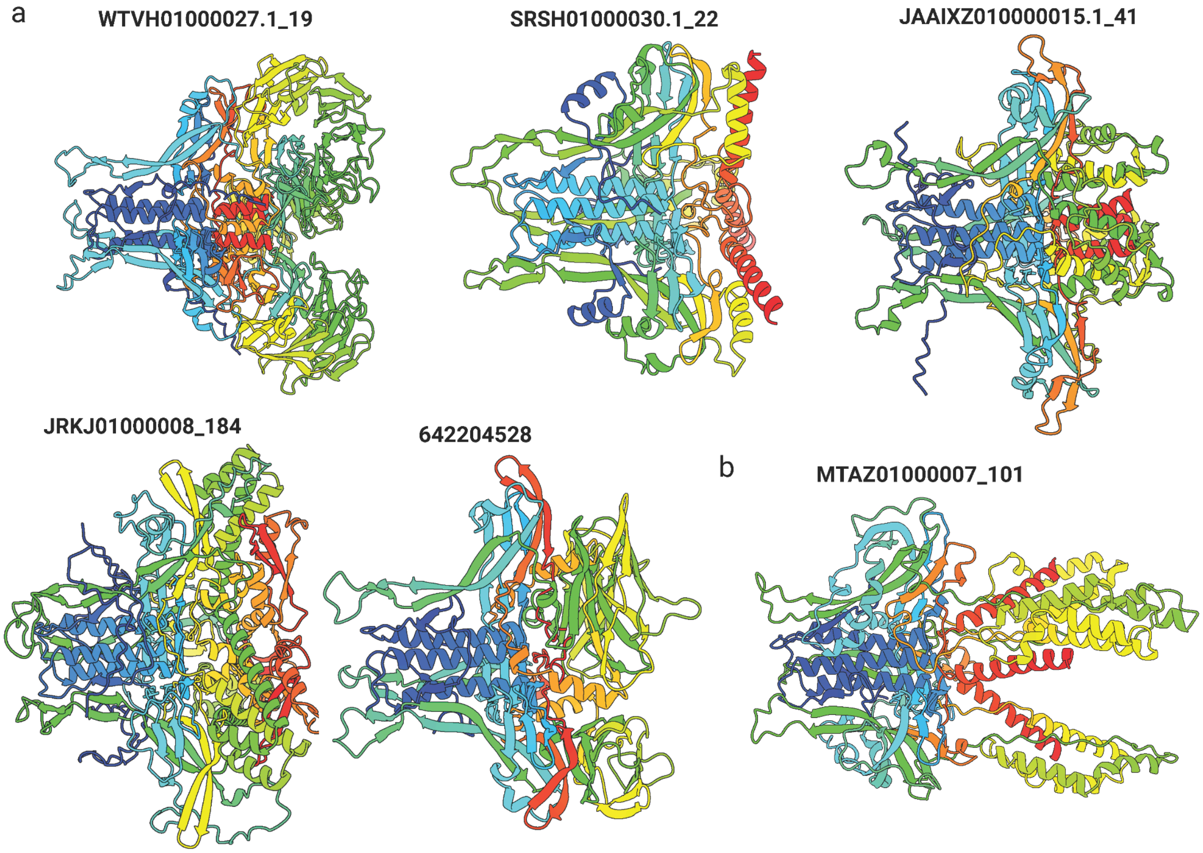

Supplementary Figure 7. Shoulder domains tend to get intertwined with adjacent structural features

Examples for eBAP3 **(a)** and eBAP4 **(b)** intertwining with downstream adjacent structural features. We used a rainbow diagram to demonstrate the fold's back bone being included in the adjacent domain then going back and serving as beta-strands incorporated in the "shoulders" region typical to eBAP3-5. We observed that this trait, speculated to enhance structure stability, also enhances divergence in these protein families.

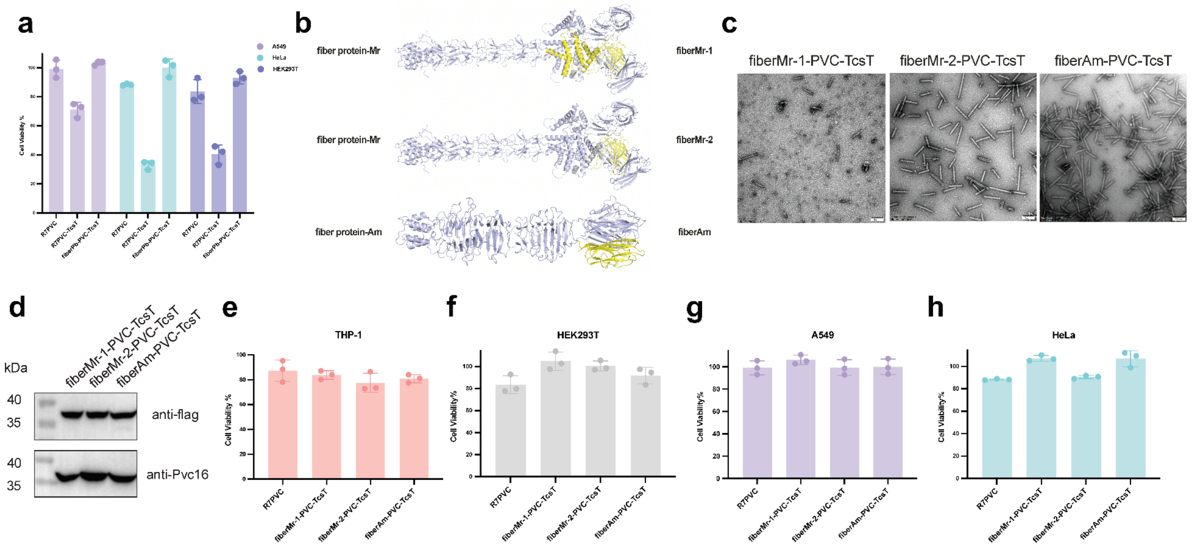

Supplementary Figure 8. Target recognition by screened eCIS tail fibers.

(a) Cell orientation of the fiberPb-modified PVC complex. Killing of A549, HeLa, and HEK293T cells by PVC complexes at 0.5 mg/mL concentration after 48h. Empty R7PVC was used as control.

(b) The possible fiber fragments in the predicted fiber proteins. Yellow parts represent the possible fiber fragments. Mr, Mycetohabitans rhizoxinica HKI 454; Am, Aquimarina sp. AU119.

(c) Western blotting validation of engineered PVC assembly and protein loading. (D) The NSEM observation of engineered PVC particles. Scale bar, 100 nm. **(E-H)** Killing of THP-1, HEK293T, A549, and HeLa cells by engineered PVC complexes at 0.5 mg/mL concentration after 48h. Empty R7PVC was used as negative control.

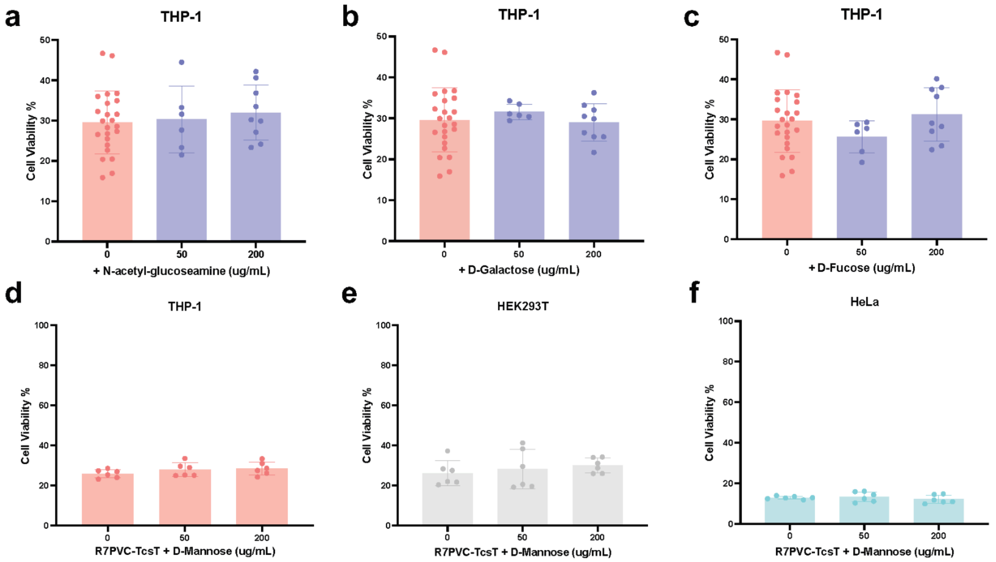

Supplementary Figure 9. Verification of glycans' effects on fiber-cell recognition.

(a-c) Glycans that cannot inhibit the fiberPb-PVC-cell recognition. Killing of THP-1 cells by 0.5 mg/mL PVC complexes pretreated with N-acetylglucosamine, D-Galactose or D-Fucose after 48h.

(d-f) D-Mannose cannot inhibit the cytotoxicity of R7PVC-TcsT. Killing of THP-1, HeLa, and HEK293T cells by 0.5 mg/mL R7PVC complexes pretreated with D-Mannose after 48h.

a

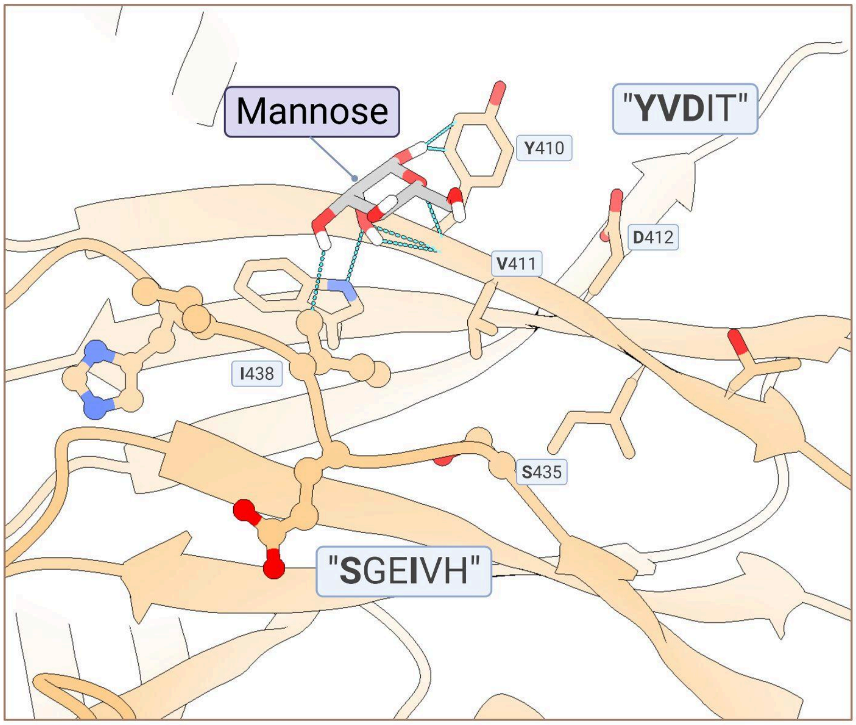

b

>FiberPb 406-572

IVSG**YVDIT**IDPFT
KIAVSPFGRAMKSF
Y**SGEIVH**GLGEGNV
LIITSLEERSGDAF
SDILASGDRVYGGA
SEVFKGSEFEPDAP
EVKIGTIAYPQKGT
FRIGVKVQQSSEQS
TVRVRWWAYSAEMA
SSVSSSGNSGDSLD
EALSAMREAASAGS
DVE

alpha-helix beta-sheet

Supplementary Figure 10. Molecular docking of D-mannose to fiberPb binding domain. **(a)** 3D structure showing D-mannose (sticks) docked to the fiberPb receptor-binding domain with binding pocket residues highlighted, demonstrating a probable participation of previously discovered conserved motifs (shown in grey boxes) in target glycan binding **(b)** Amino acid sequence with secondary structure coloring (α-helices: blue, β-sheets: yellow, loops: uncolored, motifs: bolded) and predicted binding motifs in bold (YVDIT, SGEIVH). Created in BioRender. Levy, A. (2025) https://BioRender.com/q2rg02f

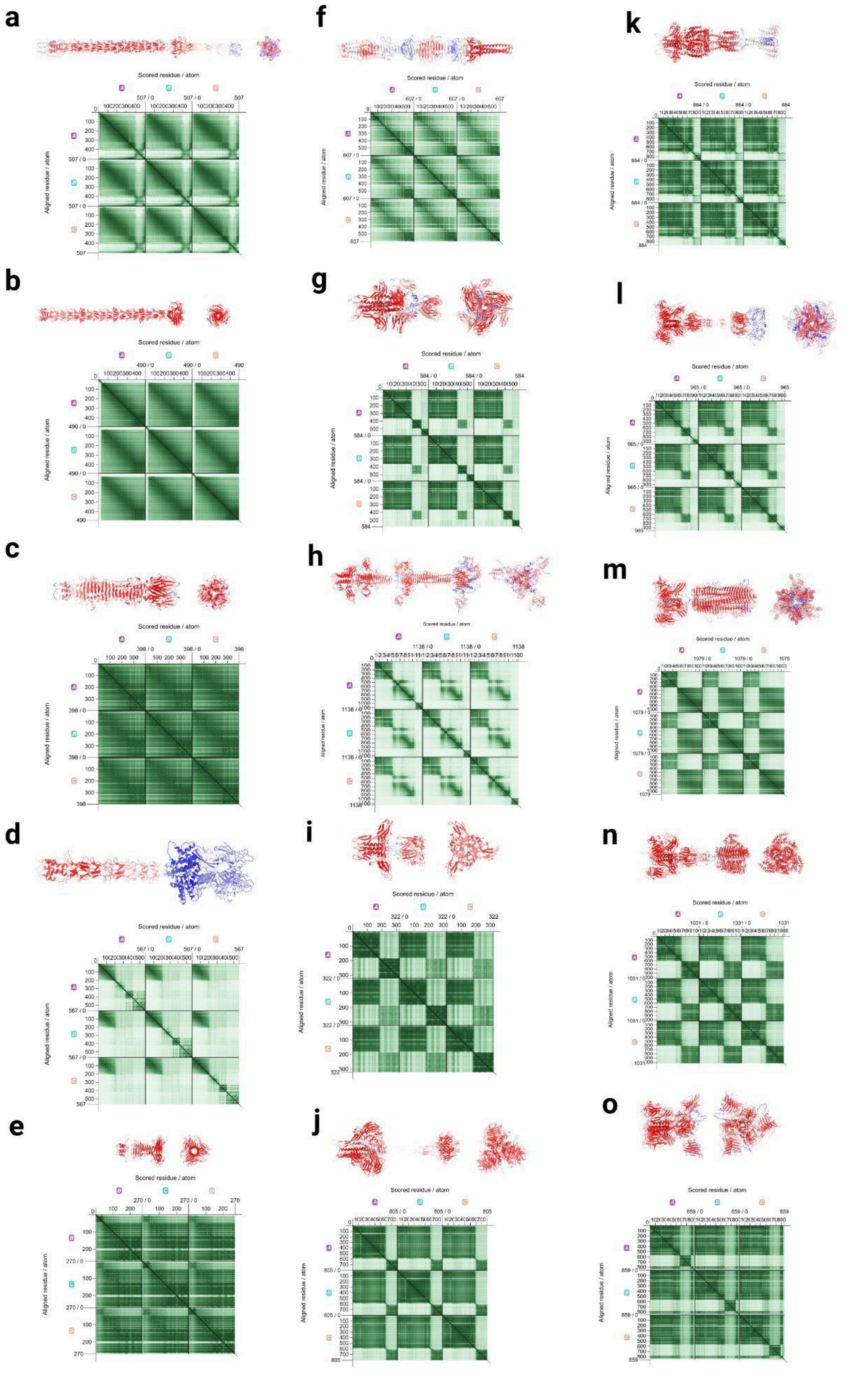
a
b
c
d
e
f
g
h
i
j
k
l
m
n
o
Scored residue / atom
Aligned residue / atom

Supplementary Figure 11. Structural diversity and model confidence for eCIS tail fiber proteins. Panels **(a–o)** display representative AlphaFold2-predicted trimeric structures for all major eCIS tail fiber groups characterized in this study. Each panel shows, at the top, the structural model colored by per-residue pLDDT confidence score (blue: low, red: high), and, at the bottom, the Predicted Aligned Error (PAE) plot reflecting inter- and intra-domain prediction reliability. Panels **(a)** and **(b)** present canonical Pvc13 and Afp13 fibers, while panel **(c)** highlights the C1q fiber as an example of horizontal gene transfer, specifically showing the adenovirus shaft from Mycetohabitans rhizoxinica HKI 454 (IMG gene ID: 650723730). Panel **(d)** introduces an eBAP2 mini-fiber from Derxia gummosa DSM 723 (2529305320), and panel **(e)** shows the C1q fiber from Aquimarina sp. AU119 (2606531960) and an S74 domain fiber from Microscilla marina ATCC 23134 (2639240252). In panel **(f)**, we feature the eBAP3 C3b/BIG domain from isolate An92 sp002159175 (NFGZ01000037_8); panel **(g)** presents an SLL lectin from Pseudoxanthomonas broegbernensis (2861529714); and panel **(h)**, another SLL lectin from Embleya scabrispora (KB889561.1_365). Panel **(i)** shows a beta-propeller/collagen module from Aromatoleum buckelii (WTVH01000027.1_19), and panel **(j)** highlights an eBAP4 bulky shaft from Cellulomonas sp000688475, with panel **(k)** displaying a helical extension from Malonomonas rubra (2588100597). Panels l through o summarize structural diversity within eBAP5: **(l)** presents a macroglobulin and lectin-like domain from Desulfococcus multivorans (ATHJ01000059_23); **(m)** features a mega pectin lyase from Rhizobium grahamii (2535518109); **(n)** includes a macroglobulin and pectin lyase from Pseudogulbenkiania ferrooxidans A (644377952); and **(o)** shows a hemagglutinin domain from Thioflavicoccus mobilis (2507114133). The figure was created in BioRender. Levy, A. (2025) https://BioRender.com/q2rg02f
