# Supplementary material for: A comprehensive catalogue of receptor-binding domains in extracellular contractile injection systems
Source: Nat Commun. 2026 Jan 22;17:1939. doi: 10.1038/s41467-026-68710-y (PMC12923769; doi:10.1038/s41467-026-68710-y)
Supplement: Supplementary file 2 — Description of Additional Supplementary Files [file 41467_2026_68710_MOESM2_ESM.pdf]

Description of Additional Supplementary Files:

Supplementary Data 1. eCIS- and T6SS-associated PFAM domains.

Supplementary Data 2. Genes of eCIS clusters (predicted operons) in GFFformat representing genomic positions of each gene within the predicted operons. Each row represents a gene. ‘genomeNumber’ is IMG genome ID (from https://img.jgi.doe.gov/cgibin/m/main.cgi) and ‘geneID’ represents an IMG gene ID. seqID is a contig or scaffold within the genome.

Supplementary Data 3. eBAP1 information: cluster ID, genome ID, gene ID.

Supplementary Data 4. eBAP2 information: cluster ID, genome ID, gene ID.

Supplementary Data 5. eBAP3 information: cluster ID, genome ID, gene ID.

Supplementary Data 6. eBAP4 information: cluster ID, genome ID, gene ID.

Supplementary Data 7. eBAP5 information: cluster ID, genome ID, gene ID.

Supplementary Data 8. Fiber gene candidates for PVC engineering.

Supplementary Data 9. Plasmids and strains used in this study.

Supplementary Data 10. fiberPb wild-type and mutant sequences.

Supplementary Data 11. Genome sources of the data analyzed. PATRIC is the Comprehensive Bacterial Bioinformatics Resource with a Focus on Human Pathogenic Species. Genomes that start with GCA are from NCBI, “blackwell” is assembled genomes from Blackwell et al. paper: https://journals.plos.org/plosbiology/ar ticle?id=10.1371/journal.pbio.3001421

Supplementary Data 12. Alphafold2 database metrics of the different proteins.
