# Supplementary material for: A comprehensive catalogue of receptor-binding domains in extracellular contractile injection systems
Source: Nat Commun. 2026 Jan 22;17:1939. doi: 10.1038/s41467-026-68710-y (PMC12923769; doi:10.1038/s41467-026-68710-y)
Supplement: Supplementary file 3 — Supplementary Dataset 1-12 [file 41467_2026_68710_MOESM3_ESM.zip › Supplementary Datasets/Supplementary Data Legend.docx]

## **Supplementary Data Legend**

**Supplementary Data 1: eCIS, T6SS related Pfams**
This file contains a comprehensive list of Pfam domains used to computationally identify and distinguish extracellular Contractile Injection System (eCIS) operons from Type VI Secretion System (T6SS) loci. The table includes: Pfam accession numbers, domain names, functional categories, scoring weights assigned during the computational search (with higher weights for distinctive eCIS markers such as DUF4157, Pvc16N, and CIStube), and exclusion criteria Pfams specific to T6SS systems.

**Supplementary Data 2: eCIS_clusters_gff_format**
This file provides the complete genomic identifiers and tags for all initial 3,719 identified eCIS loci in GFF (General Feature Format) format (gene cluster may be with or without identified fibers). Each entry includes: assigned cluster ID, genome ID and gene IDs for all clusters. This dataset serves as the main index for our work.

**Supplementary Data 3: eBAP1_clust_genome_gene**
This file documents all 362 tail fiber genes containing the eBAP1 (eCIS Baseplate Anchor Protein 1) domain. The table includes: cluster ID, gene IDs, genome accession numbers and locus tag. ​

**Supplementary Data 4: eBAP2_clust_genome_gene**
This file provides comprehensive information for all 603 tail fiber genes harboring the eBAP2 domain. Each entry contains: cluster ID, gene IDs, genome accession numbers and locus tag.​

**Supplementary Data 5: eBAP3_clust_genome_gene**
This file documents all 1,420 tail fiber genes containing the eBAP3 domain (characterized by a 200-amino-acid region with distinctive "shoulders" structural feature). The comprehensive table includes: cluster ID, gene IDs, genome accession numbers and locus tag.​

**Supplementary Data 6: eBAP4_clust_genome_gene**
This file provides detailed characterization of 402 tail fiber genes containing the eBAP4 domain, which corresponds to the first 200 amino acids of tCIS (tailocin Contractile Injection System) crown genes. The table includes: cluster ID, gene IDs, genome accession numbers and locus tag.​

**Supplementary Data 7: eBAP5_clust_genome_gene**
This file documents all 658 tail fiber genes in the eCIS dataset containing the eBAP5/DUF6519 domain, along with an expanded dataset of 1,114 additional genes from the InterPro database. Each entry includes: cluster ID, gene IDs, genome accession numbers and locus tag.​

**Supplementary Data 8: fiber gene candidates.xlsx**This Excel file lists the tail fiber genes selected for experimental validation and engineering into PVC chassis. The spreadsheet contains: gene locus tag and features for fiber-Pb (*Paenibacillus* sp. URHA0014), fiber-Mr, and fiber-Am.

**Supplementary Data 9: plasmids and strains.xlsx**
This Excel file contains documentation of all molecular biology reagents used in this study. The spreadsheet includes multiple tabs for: Plasmid constructs with names (pCNM3-fiber variants, pBR-LysR, pBBRN-TcsT), creation source and usage during this study. Additionally, western blot antibodies are displayed as well.

**Supplementary Data 10: fiber wt and mutants seq.xlsx**
This Excel file documents all wild-type and mutant fiber sequences used in site-directed mutagenesis experiments. The spreadsheet includes: complete nucleotide and amino acid sequences for wild-type fiber-Pb; all mutant variants (VDIT motif mutations, IVF/IDF motif mutations, K5 lysine mutations); primer sequences used to introduce each mutation with restriction sites and overhangs.

**Supplementary Data 11: main database genome metadata.xlsx**
This Excel file provides comprehensive metadata for all bacterial genomes in our basal database used for searching eCIS operons. The spreadsheet includes: genome IDs and source database; environmental metadata; taxonomic classification (phylum, class, order, family, genus, species); ecological metadata including isolation source (soil, aquatic, host-associated, clinical).

**Supplementary Data 12: Alphafold2 database metrics**This file contains quality metrics and structural prediction statistics for all representative tail fiber protein structures predicted using AlphaFold2-multimer. The table includes: fiber cluster IDs corresponding to proteins clustered at 70% sequence similarity; protein chain length, predicted local distance difference test (pLDDT) scores for overall structure confidence; predicted pTM and ipTM -scores.
